# Risk Assessment and Implications of Schoolchildren Exposure to Classroom Heavy Metals Particles in Jeddah, Saudi Arabia

**DOI:** 10.3390/ijerph16245017

**Published:** 2019-12-10

**Authors:** Mansour A. Alghamdi, Salwa K. Hassan, Noura A. Alzahrani, Fahd M. Almehmadi, Mamdouh I. Khoder

**Affiliations:** 1 Department of Environmental Sciences, Faculty of Meteorology, Environment and Arid Land Agriculture, King Abdulaziz University, P.O. Box 80208, Jeddah 21589, Saudi Arabia; fmehmadi@gmail.com (F.M.A.); mkhader@kau.edu.sa (M.I.K.); 2 Air Pollution Department, National Research Centre, El Behooth Str., Dokki, Giza 12622, Egypt; salwakamal1999@gmail.com; 3 Office of Education/South Jeddah (Girls), Department of Primary Grades, Ministry of Education, Jeddah 23524, Saudi Arabia; naz1407@hotmail.com

**Keywords:** classrooms air conditioner filter particles, heavy metals, contamination level, indoor air quality, health risk, schools

## Abstract

Classrooms Air Conditioner Filter (CACF) particles represent all of the exposed particles that have migrated to the interior environment. This study was conducted to assess the heavy metals contamination in CACF particles from Jeddah primary schools located in urban, suburban and residential areas; and to evaluate their health risks of children exposure (non-carcinogenic and carcinogenic). Heavy metals levels in CACF particles of schools were in the following order: urban schools > suburban schools > residential schools. Fe, Mn and Zn were the dominant species. Geo-accumulation index (I_geo_), contamination factor (CF) and pollution load index (PLI) values indicated that the contamination levels was in the following order Cd > Pb > Zn > As > Cu > Ni > Mn > Cr > Co >V > Fe. School CACF particles was moderately contaminated with As and Zn and moderately to heavily contaminated with Pb and Cd. Enrichment factors (EFs) indicated that Zn, Cd, Pb, As and Cu in CACF particles were severe enriched. The hazard quotient (HQs) and hazards index (HI) values for heavy metals were lower than the acceptable level of one. As, Pb, Cr and Mn were exhibited high non-cancer effects for children. The lifetime cancer risk (LCR) and total lifetime cancer risk (TLCR), HQs and HI values for the different exposure pathways of heavy metals decreased in the following order: ingestion > dermal contact > inhalation. Carcinogenic and non-carcinogenic risk rank order of schools were urban schools > suburban schools > residential schools. The LCR and TLCR of heavy metals was in the following order: Co > Ni >Cr > Cd > As > Pb. The ingestion lifetime cancer risk (LCR_ing_) and TLCR values from exposure to Ni and Cr in urban and suburban schools, Cd in urban schools, and Co in all Jeddah schools only exceed the acceptable range (1 × 10^−6^–1 × 10^−4^) Only LCR_ing_ and TLCR values from exposure to ∑ carcinogens exceed the acceptable level.

## 1. Introduction

Air conditioning (AC) is widely used as an effective mean to control the heat and to keep the indoor air quality within safe levels, since air pollutants such as particulate matter can be captured on the AC filter [1]. These deposited particles refer to the particles that settle on the AC filter during air current impact; their particle size is assumed to be less than 100 µm [2]. On the other hand, AC filter particles can considered as a source of particles, organic and inorganic contamination that was deposited on these filters [3]. Particles on an AC filter can be resuspended and adhere onto interior surfaces, and then building occupants, including children, can be exposed to ingest them [4]. Therefore, particles that accumulate on the AC filter will represent all of the exposed particles that have migrated to the interior environment, including suspended and resuspended settled particles during occupant activities. Skin, hair, smoking, cooking and heating emissions, construction and building materials, clothing, furnishing and other materials found in building interiors are the interior particulate sources [5,6], whereas the particulates that penetrate through doors, ventilation system, windows and AC filters for fresh air are considered the exterior particulate sources [7].

Interior particles’ composition differs from site to site, depending on the interior and exterior activities. Several studies have reported that interior particles contain heavy metals and other toxic materials [8,9,10,11,12] that make them one of the most important contaminants because they have a negative impact on human health, especially young children. The interior particles’ heavy metals sources are varied, depending on the location of the building, conditions, the activities occurring in the interior environment as well as exterior sources [8,13,14]. Cigarette smoking, construction, cosmetics and carpet materials, demolition, leaded paints, etc. all elevate the interior levels of chromium (Cr), zinc (Zn), copper (Cu), manganese (Mn), lead (Pb), and cadmium (Cd) [15,16,17,18,19]. Many studies have concluded that the road dust heavy metals from heavy traffic are another source of heavy metals in the interior environment [20,21,22,23]. Cd, Cu, Pb, nickel (Ni) and Zn pollutants are emitted from fossil fuel and coal combustion [19,24]. Lubricating oil use elevates aluminum (Al), Mn, magnesium (Mg) and iron (Fe) levels in particles [25]. Traffic and industrial emissions, surface soil, building materials, particularly from renovation process are the sources of preschools’ heavy metals [26]. Classroom particles from schools located in industrial vicinities and heavy traffic areas contain high levels of heavy metals [27,28].

It has been reported that exposure to Pb, Cd, Hg, Zn and Cr leads to several diseases like kidney failure, cardiovascular, blood and bone diseases, nervous system and gingivitis [29,30]. Heavy metals like Fe, As, and Pb of interior particle sources can cause cancer and cardiac injury [31]. Accumulation of metals in human tissues and other parts of the body will probably result in metal-associated diseases later in life [32]. Inhalation, dust ingestion, and dermal contact are the common pathways whereby heavy metals enter the body [33,34]. In general, oral ingestion is the main pathway exposure to these heavy metals in contaminated particles [35,36,37]. Children in particular are more exposed to interior particles due to their tendency to play on the ground and put things in their mouth [38].

Air quality inside schools, particularly in primary schools, is very important to children due to their higher inhalation rates per body mass, long time spent in schools, and higher sensitivity of children to environmental pollutants. Exposure to even low concentrations of air pollutants in schools leads to various health complaints, loss of productivity, effects on the academic performance [39] and the mental stability of children [40]. School classroom particles’ heavy metals adversely affect students’ memory potential [41]. Hence, the contamination and health risks related to the Classrooms Air Conditioner Filter (CACF) particles need to be evaluated. Therefore, CACF particles that representing the suspended and resuspended settled particles can provide useful information about air quality in the interior school environment and the possible hazards resulting from the exposure of children to classrooms environments with heavy metals particles. Thus, the present study aimed to assess the heavy metal contamination levels in CACF particles at selected Jeddah primary schools; and to evaluate the resulting health risks (non-carcinogenic and carcinogenic) to children due to this exposure, using the United States Environmental Protection Agency (USEPA) risk assessment models.

## 2. Materials and Methods

### 2.1. Study Area

**Figure 1:**
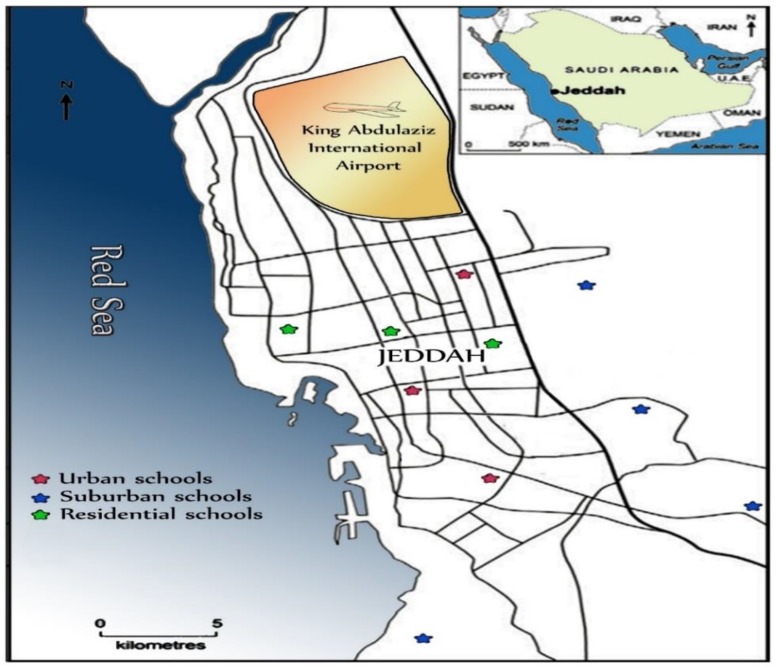
Map of Jeddah showing sampling site distribution in the different functional areas.

Jeddah (latitude 29.2 North and longitude 39.7 East) is the largest city in Makkah Province and the second largest city in Saudi Arabia after the capital Riyadh (Figure 1). The city is traditionally known as “The Red Sea Bride” as it is located on the east coast of Red Sea, with an urban area of some 1765 km^2^ and more than 4 million inhabitants (2016 estimates) [42]. The city is surrounded by mountains to the northeast, east and southeast. The international airport is located in the northern part of the city and the harbor located in the south-western part of the city; they are heavily used during certain seasons of the Hijri calendar such as Omra and Hajj. South of Jeddah city are the industrial sector and a naval port.

The weather in Jeddah is hot in summer, and moderate in winter without a real cold season. The average high temperatures in July and January are 39.4 °C and 29 °C, respectively, with scarce precipitation and high humidity. Calm to moderate winds are blown to the city from the northwest for most periods of the year. Historically, it recognized as the gate of Makkah city where the pilgrims land in Jeddah airport before going to the holy city. The city is modern and distinct, with many economic, industrial and tourism activities that made Jeddah an attractive place for business, tourists and travellers alike. Besides that, huge public projects have been recently built: a new airport, railway stations and waterfront that will emphasize the city’s transformation in the new era. Road traffic is more than 1.4 million vehicles, fueled mainly by unleaded gasoline and diesel and stationary sources, like oil refining, seaport activities, a desalination plant, power-generation plants and industrial activities in the south, are the main emission sources of air pollutants in the city.

In Saudi Arabia, dust storms is considered to be one of the most severe environmental hazards. Due to the topography, drought, light textured topsoil and scant vegetative cover, Saudi Arabia is susceptible to dust and dust storms. Previous studies revealed that the highest incidence of dust storms frequency events occurred during the spring in Saudi Arabia [43,44,45]. The sand-dust events during the spring season carry more coarser than fine particles to Jeddah and the soil originated species contribute mainly to dust storm particles [44]. During the period of the present study, dust storms occurred in April and May 2019. Although there is no monitoring station or sensors next to each school or published data on the concentration levels of particulate matter in the atmosphere of the present study areas during the period of study, a recent study on particulate matter (PM_2.5_, PM_2.5–10_, and PM_10_) in the atmosphere of an urban area of Jeddah indicated that the highest seasonal averages concentrations were found in spring, whereas the lowest values were found in autumn [46].

### 2.2. Sample Collection and Preparation

CACF particle samples were collected from 10 primary schools located in urban, suburban and residential areas (Figure 1), representing different environmental conditions, and various functional categories to reveal the pollution impacts resulting from various human activities. The sampling period was predefined to obtain maximum loadings of particulate matter during two months of the spring season. In the present study, 40 samples were collected, during the months of April–May 2019. At each school, four samples were collected simultaneously from different classrooms from the air conditioner filters of each classroom, which were generally window air conditioners with 42 × 36 cm size filters. The samples were collected using a plastic brush, clean polythene sheets and airtight polyethylene bags. To ensure proper collection of CACF samples, the particles deposited on CACF materials were extracted in a closed room. The CACF particles were trapped slowly in order to avoid the interference of air, as the deposited particles on the CACF are of small size and as such they can become resuspended with ease [47]. The collected samples were stored in clean-labelled polyethylene bags and transported to the lab. CACF particles samples were air-dried at room temperature in the laboratory, and then the coarse impurities were removed using a 1.0 mm mesh nylon sieve. The rest of the samples were homogenized and sieved through a 38-μm sieve size and stored in small self-sealing plastic bags for analysis. In the present study, CACF particles with particle size ≤38 µm diameter was selected to determine their heavy metal concentrations because: (1) the concentration of metal increases with the decrease in dust particle size [48], (2) they represent high health risks [49,50] and (3) they remain airborne for long durations and can easily be transported [35,51].

### 2.3. Sample Digestion and Analysis

To measure the concentrations of heavy metals in CACF particles, accurately weighed dust samples (1 g) were digested in an acid (nitric and hydrochloric) mixture using the method described by Shabbaj et al. [37]. After digestion, sample solutions were filtered through Whatman filter paper (No. 42), and diluted to 100 mL with deionized water. Samples were stored at 4 °C in pre-cleaned polyethylene bottle until analysis. Inductively Coupled Plasma Optical Emission Spectrometry (ICP- OES-5100, Thermo Fisher Scientific, MA, USA) was used to determine the concentrations of the heavy metals (Fe, Mn, Zn, Pb, Cd, V, Co, Ni, As, Cr and Cu) in the digested CACF particle samples. The quality of data was ensured using standard materials between samples. The standard deviation of repeated measurements of standards was used to determine the precision of measured metals; it was less than 2.5%. Heavy metal concentrations in laboratory blanks, filter blanks and reagent blanks were determined by the same procedure described above in order to assess external heavy metal pollution resulting from the analytical procedures. No contamination was noted.

### 2.4. Pollution Assessment Methodology

#### 2.4.1. Contamination Factor (CF)

Assessment of the level of contamination in CACF particles by heavy metals is expressed in terms of a contamination factor (CF). The CF for each heavy metal was calculated as the ratio between the metal concentrations in CAC filter particles with its background value [52]:

(1) $$\mathrm{CF}=\frac{C_{n}\;Sample}{C_{n}\;Background}$$

where CF < 1 refers to low contamination, 1 ≤ CF > 3 indicates moderate contamination, 3 ≤ CF ≤ 6 means considerable contamination and CF > 6 refers to very high contamination [53].

#### 2.4.2. Pollution Load Index (PLI)

PLI for each heavy metal in CACF particles samples was calculated using the following equation [54]:

(2) $$\mathrm{PLI}={\left({\mathrm{CF}}_{1}\times {\mathrm{CF}}_{2}\times {\mathrm{CF}}_{3}\times {\mathrm{CF}}_{4}\times \ldots \times {\mathrm{CF}}_{n}\right)}^{1/n}$$

where PLI is the pollution load index, CF is the contamination factor calculated as described in Equation (1) and n is the number of heavy metals studied. A PLI value < 1 indicates no pollution, PLI = 1 present that only baseline levels of pollutants are present, whereas PLI value > 1 indicates polluted CACF particles [54].

#### 2.4.3. Geo-Accumulation Index (I_geo_)

The heavy metal contamination levels in CACF particles was evaluating by using the geo-accumulation index (I_geo_) which is widely applied to assess heavy metal pollution in dust [55]. Seven different enrichment classes (Table 1) ranging from (0–6), starting from “normal background value” to “very heavily polluted” were used to evaluate the heavy metal pollution [56,57]. The I_geo_ was calculated from the following equation [58]:

(3) $$I_{\mathrm{geo}}=\log_{2}\left(C_{n}/1.5B_{n}\right)$$

where I_geo_ is the geo-accumulation index for different heavy metals, *C_n_* the measured concentration of the heavy metals in CACF particles samples. The constant 1.5 was used to minimize the effect of possible variations in the background values. While the *B_n_* refers to the background value of metals in the earth’s crust [59].

#### 2.4.4. Enrichment Factor (EF)

EF is used to distinguish among the anthropogenic sources and natural origin of heavy metals in CACF particles, as well as, to estimate the degree of the anthropogenic contribution and metal contamination. It was calculated using Equation (4) [60,61]:

(4) $$\mathrm{EF}=\frac{{\left(C_{x}/C_{\mathrm{reference}}\right)}_{\text{Road dust}}}{{\left(C_{x}/C_{\mathrm{reference}}\right)}_{\text{Earth crust}}}$$

where EF refers to the enrichment factor for each heavy metal, C_x_ is the target heavy metal concentrations, and C_reference_ is the reference metal concentration. In the present study, Fe was selected as a reference metal that used for the calculation of EF, since our calculation of EFs was done assuming that contributions of man–made sources to Fe are insignificant in Jeddah. The composition of the Earth’s crust was taken from Taylor [59] and Taylor and McLennan [62], since average local soil profile data was not available and will be investigated in the follow up study. The use of average crust values provides a meaningful comparison to other studies that commonly use this technique. EF values less than 2 refer to a deficiency to minimal enrichment [63], between 2 and 10 indicate moderate enrichment, whereas higher than 10 shows severe enrichment [64].

### 2.5. Health Risk Assessment (HRA)

HRA models, developed by the United States Environmental Protection Agency (USEPA), were used to quantify the health risk (carcinogenic and non-carcinogenic) for children from exposure to heavy metals in CACF particles [65,66]. Children in school classrooms are exposed to heavy metals in CACF particles through direct ingestion, inhalation and dermal absorption. The total non-carcinogenic risk for children was calculated for each heavy metal in CACF particles by the summation of the individual risks that calculated from the three exposure pathways [65,67].

Daily dose (ADD) (mg kg^−1^ day^−1^) average from exposure to heavy metals in CACF particles through the different exposure pathways was calculated according to Exposure Factors Handbook [68] and the Technical Report of USEPA [69] using the following equations:

(5) $${\mathrm{ADD}}_{\mathrm{ing}}=\frac{C\times \mathrm{IngR}\times \mathrm{CF}\times \mathrm{EF}\times \mathrm{ED}}{\mathrm{BW}\times \mathrm{AT}}$$

(6) $${\mathrm{ADD}}_{\mathrm{inh}}=\frac{C\times \mathrm{InhR}\times \mathrm{EF}\times \mathrm{ED}}{\mathrm{PEF}\times \mathrm{BW}\times \mathrm{AT}}$$

(7) $${\mathrm{ADD}}_{\mathrm{dermal}}=\frac{C\times \mathrm{SA}\times \mathrm{CF}\times \mathrm{AF}\times \mathrm{ABF}\times \mathrm{EF}\times \mathrm{ED}}{\mathrm{BW}\times \mathrm{AT}}$$

(8) $$\mathrm{HQ}=\frac{\mathrm{ADD}}{\mathrm{RfD}}$$

(9) $$\mathrm{HI}={\mathrm{HQ}}_{\mathrm{ing}}+{\mathrm{HQ}}_{\mathrm{inh}}+{\mathrm{HQ}}_{\mathrm{dermal}}$$

(10) LCR=ADD×SF

(11) $$\mathrm{TLCR}={\mathrm{LCR}}_{\mathrm{ing}}+{\mathrm{LCR}}_{\mathrm{inh}}+{\mathrm{LCR}}_{\mathrm{dermal}}$$

where the ADD_ing_, ADD_inh_ and ADD_dermal_ are the daily dose (mg kg^−1^ day^−1^) average exposure to heavy metals through ingestion, inhalation and dermal contact, respectively. RfD and SF are the values of reference dose (mg kg^−1^ day^−1^) and slope factor [36,70,71,72,73]. The detailed description about the values of exposure factors for children and adults that were applied to the above model Equations (5)–(11) in the present study are given in Table 2. In order to evaluate the non-carcinogenic hazards from exposure to heavy metals in CACF particles in Jeddah schools, HQ (hazard quotient), and HI (hazards index) Equations (8) and (9) were applied [74,75].

For carcinogenic risk, the lifetime cancer risk (LCR) of children from potential carcinogen exposure in CACF particles over a lifetime was calculated using Equation (10), for ADD and SF is the slope factor for cancer. Total lifetime cancer risk (TLCR) is the summation of LCR calculated for ingestion, dermal contact and inhalation [12] using Equations (10) and (11).

There is a chance that adverse health effects occur when HQ and total risk of non-carcinogenic (HI) more than one for a single metal, and the probability increase with increasing the HI value [70,71]. On the other hand, the TLCR values that exceed the acceptable carcinogenic risk range (1 × 10^−6^–1 × 10^−4^) indicate that potential carcinogenic risks are occur [76].

## 3. Results and Discussion

### 3.1. Heavy Metal Concentrations in CACF Particles

**Figure 2:**
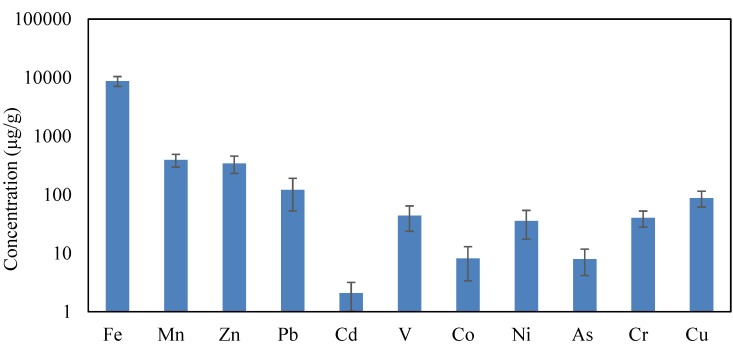
The mean concentrations of heavy metals in CACF particles of Jeddah schools.

The average concentrations of heavy metals in CACF particles collected from Jeddah schools are shown in Figure 2. The heavy metals levels were in descending order of Fe (8752 µg/g) > Mn (392 µg/g) > Zn (343 µg/g) > Pb (121 µg/g) > Cu (88 µg/g) > V (44 µg/g) > Cr (40 µg/g) > Ni (36 µg/g) > Co (8.1 µg/g) > As (7.95 µg/g) and Cd (2.1 µg/g). Pb, Cu, Mn, Zn, Co and Cd maximum permissible concentration (MPC) in soil were 100, 100, 1500, 300, 30, and 3 µg/g, respectively [89]. In the present study, only the Pb and Zn concentrations were higher than the MPC. Due to use of the white boards with erasable ink pens for teaching instead of blackboards and chalk in Jeddah schools, it is assumed that the sources of heavy metals in CACF particles mostly originated from the exterior. Traffic that circulates around the Jeddah schools is the exterior anthropogenic source of CACF particles heavy metals. Table 3 shows comparison of heavy metals concentrations in CACF particles in Jeddah schools with those found in the school classroom particles of other cities around the world. From this table, it can be seen that the heavy metal levels in school classroom particles vary extremely between the different cities in world; this may be attributed to the variation in the exterior sources such as traffic intensity, human activities, land use type and technologies used, which are the main sources of these metals in the interior microenvironments. The heavy metals levels in CACF particles from Jeddah schools were lower/higher or similar to those found in other city schools of the world. As summarized in Table 3, for example the Cu concentration in CACF particles of Jeddah schools is lower than in Malaysia (Jengka, Selangor), Iran (Bushehr Serdang), and higher than in China (Xian), Malaysia (Sri Serdang, Shah Alam, Nigeria (Ogun State). The Pb content in CACF particles of Jeddah schools is higher than in Nigeria (Ogun State), Malaysia (Shah Alam, Sri Serdang) and Iran (Bushehr) but lower than elsewhere in Malaysia (Selangor, Jengka, Selangor), and China (Xian). The Ni concentration in CACF particles of Jeddah schools is almost similar to that of China (Xian), lower than in Iran (Bushehr), Malaysia (Serdang), and higher than in Malaysia (Shah Alam) and Nigeria (Ogun State). These results back up the concept that each city school has a distinctive combination of heavy metal composition, and their variations and similarities in levels of these heavy metals in the interior particles between the world cities’ schools may not reflect the actual natural and anthropogenic diversities among various schools settings.

The spatial differences of the heavy metals concentrations in collected CACF particles from various land use areas in Jeddah are shown in Table 4. All heavy metal concentrations (except for Fe in residential schools) in CACF particles were higher in urban schools than suburban and residential schools. Based on the concentrations of ∑metals emitted from anthropogenic sources in each functional land used areas (Mn, Zn, Pb, Cd, V, Co, Ni, As, Cr and Cu), they could be classified as follows: urban schools > suburban schools > residential schools. The wide differences in the level of heavy metals among schools in various functional areas result from the difference in exterior characteristic activities that release distinct types of heavy metals in each functional area [94,95]. In the present study, the relatively higher traffic density around the urban schools than suburban and residential schools leads to an increase in the emission of heavy metal particles in the exterior and then consequently in the interior environment of urban schools, while Fe in residential schools mostly comes from natural sources. This result is in agreement with previous studies which reported that traffic emissions are the major source of heavy metal particles that be brought into school classrooms [12,14,90,93,96,97]. Heavy metals in urban street dust originate mainly from vehicular traffic emissions [36]. Vehicle exhaust, tire, brake and engine lining wear, vehicle component collisions, and using of lubricating oils increase the levels of heavy metals [79,90,98,99,100,101].

### 3.2. Pollution Characteristics of CACF Particles

**Figure 3:**
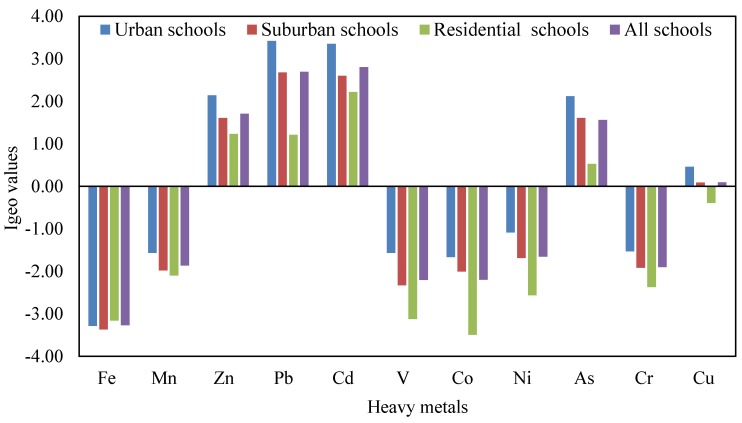
Average values of geo-accumulation index for heavy metals in CACF particles collected from schools in different areas.

Based on CF and PLI calculations, the CF and PLI values for each heavy metal in CACF particles collected from each school in various land use functional areas in Jeddah are shown in Table 5. The values of CF were different from metal to metal in all schools, with the highest Zn, Pb, Cd and As values. CF values were lower than the ones for Fe, Mn, V, Co, Ni and Cr, whereas the values were higher than the ones for Zn, Pb, Cd, Cu and As in the different schools. The lack of contamination of CACF particles of the different schools by Fe, Mn, V, Co, Ni and Cr metals, which have similar CF values of less than one, indicate that they originate from soil and resuspended dust. On the other hand, the contamination of CACF particles by Zn, Pb, Cd, Cu and As metals may be due to traffic emissions, which are the main sources of these metals in the study areas. Moreover, the highest CF values for Zn, Pb, Cd, Cu and As metals, originated from vehicle emissions, were found in CACF particles of urban schools followed by suburban and residential schools. Moreover, the CF values for the individual metal were different in urban, suburban and residential schools, with maximum values in urban schools. This may be attributed to the higher traffic density around urban schools, which lead to an increase in the concentrations of these metals in the CACF particles. Regarding the PLI values, they were 1.57, 1.12, 0.71, and 1.15 for urban, suburban, residential and all schools of Jeddah, respectively. This finding suggests that the heavy metals in CACF particles of Jeddah urban schools were more contaminated, that can be attributed to the influence of high vehicular traffic emissions around these schools. Past studies have reported that automobile emissions are the main source of heavy metals in interior particles [41,102,103,104]. The geo-accumulation index (I_geo_) categorizes the pollution levels into seven classes, depending on the severity of contamination that used to determine the metal pollution status. The I_geo_ values for heavy metals in CACF particles collected from various schools are presented in Figure 3. The order of the mean I_geo_ values in CACF particles were Pb > Cd > Zn > As > Cu > Ni > Cr > Mn > V > Co > Fe in urban schools, Pb > Cd > Zn > As > Cu > Ni > Cr > Mn > Co > V > Fe in suburban schools, Cd > Zn > Pb > As > Cu > Mn > Cr > Ni > V > Fe > Co in residential schools, and Cd > Pb > Zn > As > Cu > Ni > Mn > Cr > Co > V > Fe in all Jeddah schools. Based on the criteria of contamination of CACF particles based on I_geo_ values (Table 1), CACF particles were uncontaminated (I_geo_ class 0) by Fe, Mn, V, Co, Ni in all schools and Cu in residential schools; uncontaminated to moderately contaminated (I_geo_ class 1) by Cu in urban, suburban and all Jeddah schools and As in residential schools; moderately contaminated (I_geo_ class 2) by Zn and As in suburban schools and Zn and Pb in residential schools and Zn and As in all schools. CACF particles were moderately to heavily contaminated (I_geo_ class 3) by Zn and As in urban schools, Pb and Cd in suburban schools, Cd in residential schools and Pb and Cd in all schools, and heavily contaminated (I_geo_ class 4) by Pb and Cd in urban schools. These results indicate that CACF particles that have small particle sizes and large surface area, especially in urban schools, can carry a lot of heavy metals like Zn, As, Pb and Cd. Moreover, increases the heavy metals that are released from exterior anthropogenic sources lead to an increase in their content in school CACF particles.

**Figure 4:**
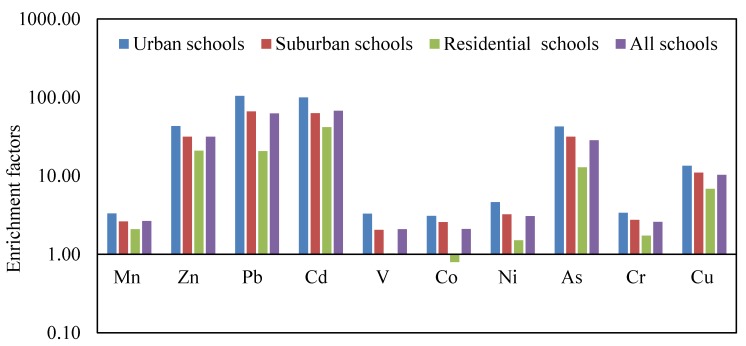
Average enrichment factors for heavy metals in CACF particles collected from schools in different areas.

The estimated value of EF for each heavy metals in CACF particles collected from schools located in various areas are represented graphically in Figure 4. EF values between 2 and 10 indicate a moderate enrichment, whereas EF values > 10 indicate severe enrichment [64]. Based on the estimated EF for each heavy metal in the present study, V, Co, Ni and Cr in CACF particles of residential schools with EF values less than 2 indicate that they were deficient to minimally contaminated and these metals originate from exterior natural sources. The EF values of Mn, V, Co, Ni, and Cr in both urban and suburban schools particles, and Mn and Cu in residential schools particles were between 2 and 10, suggesting that they were moderately enriched. Moreover, EF values larger than 10 were found for Zn, Pb, Cd, As and Cu in both urban and suburban school particles and Zn, Pb, Cd, and As in residential schools particles, indicating that they were severely enriched. With regard to all Jeddah schools, the mean values of EF for each heavy metal in CACF particles displayed the following decreasing trend: Cd > Pb > Zn >As > Cu > Ni > Mn > Cr > Co > V. For the mean EF values, Mn, V, Co, Ni and Cr were between 2 and 10, showing that they were moderately enriched. Cd, Pb, Zn As and Cu were larger than 10, suggesting severe enrichment by exterior anthropogenic sources. Cu, Cr, Ni, Pb and Zn originate from vehicle emissions [11,105]. Anthropogenic Cu and Zn are produced from traffic-related materials such as lubricating oils, vehicle brake and tire wear and yellow paint [106,107,108,109]. Enrichment of heavy metals in the interior environment may be due to exterior sources like heavy traffic roads [79,110]. Furthermore, in countries which have a desert climate, dust storms which are considered a natural source of air pollution can significantly impact interior air quality, since these storms may lead to an increase in the concentrations of metals in the exterior and consequently in the interior. A study of the effect of dust storms on the level of metals in the atmosphere of Jeddah city revealed that the dust storms lead to an increase in the concentrations of crustal elements (e.g., Si, Ca, Na, Al, Fe, K, and Mg) that originate from soil and resuspended dust, and the anthropogenic elements (e.g., Co, Cu, Zn, As, Pb, Cd, V and Ni) that are emitted from anthropogenic sources [44]. The deserts surrounding Jeddah city were evidently the sources of the mineral crust elements in CACF particles by means of regional or long range transport to the study area, beside their anthropogenic sources, while the anthropogenic heavy metals in CACF particles originate from sources found in both the local and their surrounding areas. In this study, CACF particles samples collected from the study sites represent the time of dust storms and the time of normal conditions together. Therefore, the local pollution sources in Jeddah beside the dust storms that strike Jeddah city during the sampling time (spring season) may lead to an increase the concentrations of the measured metals at all schools [44]. However, the presence and levels of heavy metals in CACF particles by means of the effect of dust storm events through regional or long range transport to the study area are not clear yet. Therefore, further studies to differentiate between the effects of dust storm events and the effects of anthropogenic activities during normal days on the heavy metal levels in CACF particles are needed. Despite the phase out of leaded gasoline usage in Saudi Arabia in 2001 [111], the enrichment of Pb in CACF particles of Jeddah schools, especially in urban areas, is still high. This may be due to historical Pb contamination of exterior road dusts and its long half-life in soils [112].

### 3.3. Health Risk (Non-Carcinogenic and Carcinogenic) Assessment of CACF Particles Heavy Metals

**Figure 5:**
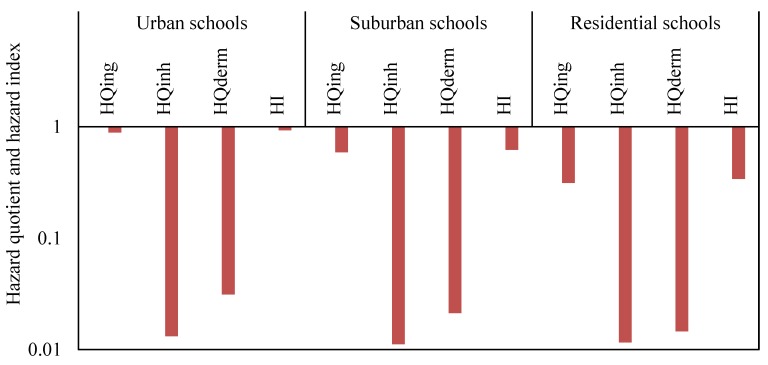
Hazard quotient and hazard index for exposure to ∑heavy metals concentrations for children in schools of different functional areas in Jeddah.

Risk characterization of the possible adverse health effects of human beings exposed to contaminants is a known as a health risk assessment [38]. Health risk (non-carcinogenic) values among children from heavy metal exposure in CACF particles collected from primary schools located in various functional areas in Jeddah through ingestion, inhalation and dermal contact are summarized in Table 6 and Figure 5. Based on the values of hazard quotient (HQ) for the ingestion (HQ_ing_), inhalation (HQ_inh_) and dermal (HQ_dermal_) pathways and hazard index (HI) from individual and total heavy metals exposure in CACF particles, the rank order of schools in different functional areas were urban schools > suburban schools > residential schools. Among the heavy metals in the various schools, As, Pb, and Fe, Mn, and Cr, V, and Pb, As displayed higher values for HQ_ing_, HQ_inh_, HQ_dermal_, and HI, respectively, compared with other metals. No significant non-carcinogenic risk for children, through ingestion, inhalation and dermal contact pathways, were found in the different schools from exposure to individual and ∑ heavy metals in CACF particles, since the values of HQs and HI for heavy metals were <1 that indicate that there was no non-carcinogenic risk [66].

**Figure 6:**
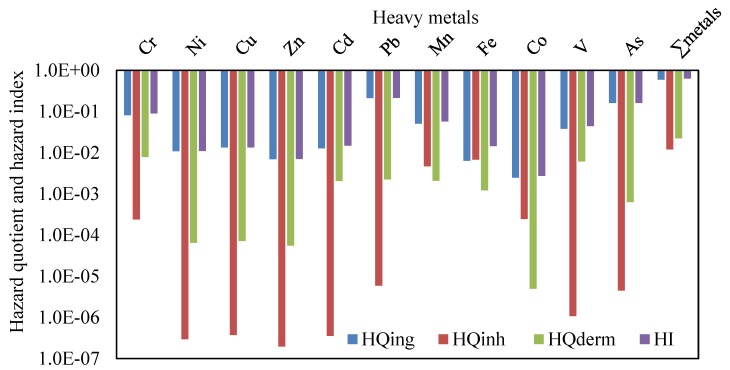
Hazard quotient and hazard index of the mean concentrations of heavy metals in all schools of Jeddah.

**Figure 7:**
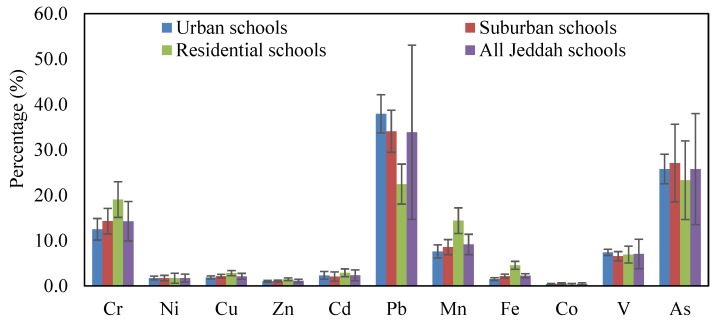
Relative contribution of individual heavy metal non-cancer to total non-cancer risks in children.

Based on the mean concentrations of heavy metals in CACF particles collected from all studied Jeddah schools, HQ_ing_, HQ_inh_, HQ_dermal_ and HI were calculated and are presented in Figure 6. The hazard quotient mean values of heavy metals were in the order of Pb > As > Cr > Mn > V > Cu > Cd > Ni > Zn > Fe > Co for HQ_ing_, Fe > Mn > Co ≈ Cr > Pb > As > V > Cu > Cd > Ni > Zn for HQ_inh_, and Cr > V > Pb > Mn > Cd > Fe > As > Cu > Ni > Zn > Co for HQ_dermal_. For HI values the order was Pb > As > Cr > Mn >V > Cd > Fe > Cu > Ni > Zn > Co. The mean relative contribution of individual heavy metal non-cancer to total non-cancer risks in the various schools are presented graphically in Figure 7. From this figure, it can be seen that Pb and As were the predominant contributors to non-cancer effects in the classrooms, and they accounted for 37.9% and 25.8% (urban schools), 34.1% and 27.1% (suburban schools), 22.5% and 13.3% (residential schools), and 33.9% and 25.8% (all Jeddah schools) of the total effects, respectively.

For the non-carcinogenic harmful effects, ingestion was the main pathway of children exposure to heavy metals particles, followed by dermal contact and inhalation. The contribution of the HQ_ing_, HQ_inh_ and HQ_dermal_ to the HI were 95.22%, 1.42% and 3.36% in urban schools, 94.78%, 1.80% and 3.42% in suburban schools, 92.30%, 3.41% and 4.29% in residential schools, and 94.6%, 1.9% and 3.5% in all Jeddah schools, respectively. This indicates that ingestion appeared to be the main route exposure of children to the heavy metals in classroom particles of Jeddah schools, consistent with other reports [17,33,34,105,113]. Tong and Lam [114] reported that dust may easily cling to the skin of children and be ingested unintentionally. In the present study, the calculated HQs and HI values for each metal and ∑HI for all the metals were lower than the safe limit of one, indicating the CACF particles pose no non-carcinogenic effect for children via the various exposure routes. The non-carcinogenic effects for the heavy metals in the present study are consistent with many studies [17,113]. Although the non-carcinogenic risk outcomes from exposure of heavy metals in CACF particles of Jeddah schools were found in the acceptable range suggested by USEPA [74], these heavy metals can cause serious health effect by its accumulation in body tissues [115,116,117]. Even if the values of HI for heavy metals are in safe level; metal exposure in high concentration can cause severe neurological, developmental health effects and increase health risks [118,119]. Sulaiman et al. [90] reported that the increasing of heavy metals might lead to the increasing of health risks values. Therefore, it can proposed that classroom particles monitoring should be done regularly to ensure lower health risks. The output of the present study may provide the initial indication of the risk for children from heavy metal exposure in classroom particles of Jeddah schools.

**Figure 8:**
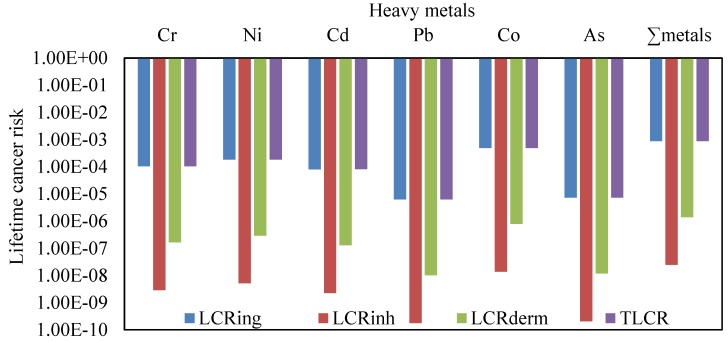
Lifetime cancer risk (LCR) of heavy metals concentrations for children in schools of Jeddah.

Cancer risk for children, from exposure of heavy metals particles (Cr, Ni, Cd, Pb, Co and As) collected from various Jeddah schools through ingestion, inhalation and dermal contact way, were calculated and are shown in Table 7 and Figure 8. Based on the values of ingestion lifetime cancer risk (LCR_ing_), inhalation lifetime cancer risk (LCR_inh_), dermal lifetime cancer risk (LCR_dermal_) pathways and total lifetime cancer risk (TLCR) from individual and ∑heavy metals exposure in CACF particles, the rank order of schools in functional areas were urban schools > suburban schools > residential schools. Among Cr, Ni, Cd, Pb, Co and As in the various schools, Co and Ni displayed higher values for LCR_ing_, LCR_inh_, LCR_dermal_, and TLCR compared with other metals. The values of LCR and TLCR were in increasing order as ingestion > dermal contact > inhalation pathways. These values were within the acceptable level (1 × 10^−6^–1 × 10^−4^), except the LCR_ing_ and TLCR values from exposure to Ni and Cr in urban and suburban schools, Cd in urban schools, and Co in all schools which exceed the acceptable range.

**Figure 9:**
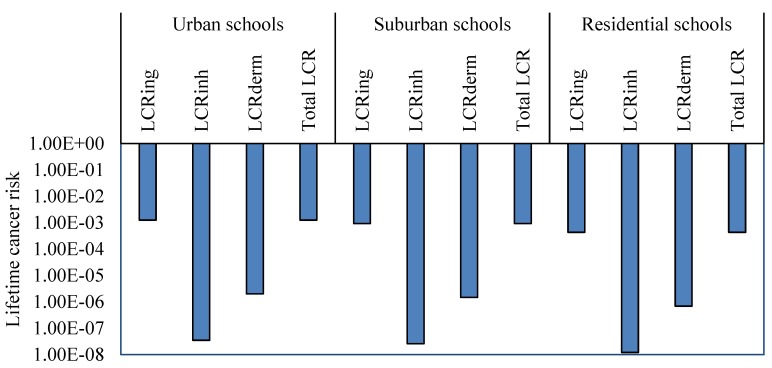
Lifetime cancer risk for exposure to ∑heavy metals concentrations for children in schools of different functional areas in Jeddah.

**Figure 10:**
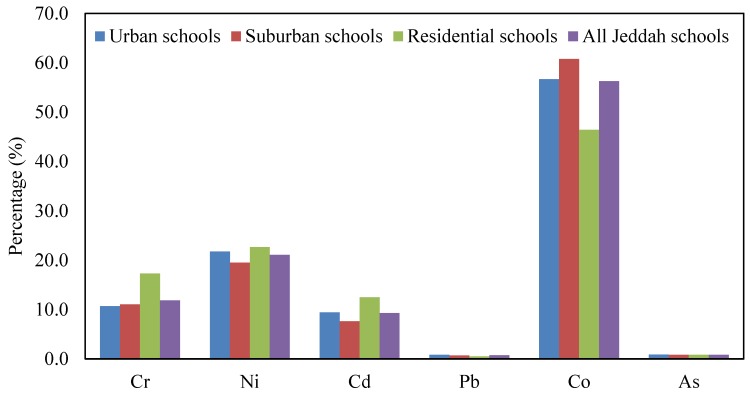
Relative contribution of individual heavy metal cancer to total cancer risks in children.

Based on the average Cr, Ni, Cd, Pb, Co and As concentrations in the CACF particles collected from all Jeddah schools, LCR_ing_, LCR_inh_, LCR_dermal_, and TLCR were calculated and represented graphically in Figure 8. The lifetime cancer risk values of heavy metals were in the order of Co > Ni > Cr > Cd > As > Pb for LCR_ing_, LCR_inh_, LCR_dermal_ and TLCR. Only LCR_ing_ and TLCR values from exposure of children to ∑carcinogens exceeded the acceptable level (Figure 9). The mean relative contribution of individual heavy metal cancer total cancer risks in different schools are represented graphically in Figure 10. From this figure, it can be shown that Co and Ni were the predominant contributors to cancer effects in the classrooms; they accounted for 56.7% and 21.8% in urban schools, 60.7% and 19.5% in suburban schools, 46.4% and 22.7% in residential schools, and 56.3% and 21.1% in all Jeddah schools of the total cancer effects, respectively. With regard to carcinogenic effects, ingestion appeared to be the main route of children exposure to Cr, Ni, Cd, Pb, Co and As in classroom particles of Jeddah schools, followed by dermal contact and inhalation. The relative contribution of the LCR_ing_, LCR_inh_ and LCR_dermal_ to the TLCR in various schools were similar, they accounted 99.84%, 0.003% and 0.16%, respectively. Similar trends were founded in previous studies [12,17,28].

## 4. Conclusions

The present study investigated the contamination and risk assessment of heavy metals in CACF particles collected from schools located in various areas in Jeddah. The levels of heavy metals in CACF particles from schools located in urban areas were higher than in those located in suburban and residential areas, which is attributed to the influence of high vehicular traffic emissions around these urban schools. Heavy metal levels in CACF particles in Jeddah schools were in descending order of Fe > Mn > Zn > Pb > Cu > V > Cr > Ni > Co > As and Cd. Based on the calculated CF and PLI values, Zn, Pb, Cd and As have the highest CF values in different schools, whereas the highest PLI value was found in urban schools, indicating more contamination. The calculation results of geo-accumulation index (_Igeo_) indicate that CACF particles were uncontaminated by Fe, Mn, V, Co, Ni in all Jeddah schools and Cu in residential schools; uncontaminated to moderately contaminated by Cu in urban, suburban and all Jeddah schools and As in residential schools. Moreover, CACF particles were moderately contaminated by Zn and As in suburban schools and Zn and Pb in residential schools and Zn and As in all Jeddah schools, and moderately to heavily contaminated by Zn and As in urban schools, Pb and Cd in suburban schools, Cd in residential schools and Pb and Cd in all Jeddah schools, and heavily contaminated by Pb and Cd in urban schools. According to the estimated EF values, heavy metals in the CACF particles of Jeddah schools demonstrated different degrees of enrichments, with Pb, Zn, Cd, As and Cu the severe enriched by exterior anthropogenic sources like traffic emissions. The HI values from exposure to CACF particles heavy metals were in the following order Pb > As > Cr > Mn > V > Cd > Fe > Cu > Ni > Zn > Co for non-carcinogenic risk, whereas TLCR values in the order of Co > Ni > Cr > Cd > As > Pb for carcinogenic risk in Jeddah schools. The major route of exposure to heavy metals that leading to both carcinogenic and non-carcinogenic risks is through ingestion followed by dermal and inhalation pathway. Co (56.3%) and Ni (21.1%) were the predominant contributors to cancer effects, whereas Pb (33.9%) and As (25.8%) were the predominant contributors to non-cancer effects in Jeddah schools. HQs and HI values from exposure to individual and total heavy metals in different schools were lower than the safe level (<1), suggesting that no non-carcinogenic risks were posed. LCR and TLCR values were within the acceptable level (1 × 10^−6^–1 × 10^−4^), except the LCR_ing_ and TLCR values from exposure to Ni and Cr in urban and suburban schools, Cd in urban schools, and Co in all schools which exceed the acceptable range. Only LCR_ing_ and TLCR values from exposure of children to ∑carcinogens exceed the acceptable level. Carcinogenic and non-carcinogenic risks rank order of schools in functional areas were urban schools > suburban schools > residential schools.

It can be concluded that the health risks might increase if the heavy metal concentrations increase too. Therefore, even if there are no significant potential health risks from exposure to heavy metals in Jeddah schools, classroom particle monitoring should be done regularly to ensure lower health risks. The output of the present study may provide an initial indication of the risk for children from heavy metal exposure in classroom particles of Jeddah schools.

## Figures and Tables

**Table 1:** Description of geo-accumulation index (I_geo_) * classes to evaluate the individual heavy metals pollution with respect to schools CACF particles quality.

I_geo_ Value (log2 (x))	I_geo_ Class	Qualitative Designation of Road Dust
I_geo_ ≤ 0	0	Uncontaminated
0 < I_geo_ ≤ 1	1	Uncontaminated to moderately contaminated
1 < I_geo_ ≤ 2	2	Moderately contaminated
2 < I_geo_ ≤ 3	3	Moderately to heavily contaminated
3 < I_geo_ ≤ 4	4	Heavily contaminated
4 < I_geo_ ≤ 5	5	Heavily to extremely contaminated
I_geo_ > 5	6	Extremely contaminated

* Wei et al. [56], Ali et al. [36].

**Table 2:** Exposure parameters used for the health risk assessment of heavy metals in school CACF particles for children through different exposure pathways.

Parameters	Symbol	Unit	Value	References
Concentration of metals in CACF particles	C	mg/kg		Present study
Ingestion rate of dust	IngR	mg/day	200	ESAG [77]; USEPA [71,72]; Van den Berg [78]
Inhalation rate of dust	InhR	m^3^/day	7.63	Li et al. [79,80]; USEPA [81]
Exposure frequency	EF	days/year	167	Peng et al. [82]; Zheng et al. [33,34]; ESAG [77]
Absorption factor (Dermal)	ABF		0.001	Wei et al. [56]; USEPA [70,71]; US Department of Energy [83]
Average body weight	BW	kg	15	Hu et al. [84]; Lu et al. [85]; Zheng et al. [33,34], ESAG [77], BMRIEP [86]
Exposure duration	ED	years	6	USEPA [81]; USEPA [70,71]
Average exposure time (non-carcinogenic)	AT	days	365 × ED	USEPA [74]
Average exposure time (carcinogenic)	AT	days	365 × 70	USEPA [74]
Conversion factor	CF	kg/mg	1 × 10^−6^	Li et al. [80]
Particular emission factor	PEF	m^3^/kg	1.36 × 109	USEPA [70,71]
Surface area of skin exposed to dust	SA	cm^2^	1600	Zheng et al. [33,34]; ESAG [77]
Skin adherence factor	AF	mg/cm^2^	0.2	USEPA [87]; Man et al. [88]

**Table 3:** Heavy metal concentrations (µg/g) in school CACF particles of different cities around the world.

Country	City	Fe	Mn	Zn	Pb	Cd	V	Co	Ni	As	Cr	Cu	Reference
Saudi Arabia	Jeddah	8751.6	391.8	342.7	121.2	2.09	43.9	8.2	35.7	8.0	40.2	87.895	This study
Malaysia	Selangor	4801		144.9	253.5						11.9		Latif et al. [11]
Malaysia	Jengka	10809		2879.0	1737.0					3.1		97.42	Sulaiman et al. [90]
China	Xi’an		565	462.6	176.2			43.3	36.2	14.5	159.7	74.2	Lu et al. [49]
Malaysia	Sri Serdang				89.1	1.89						53.27	Praveena et al. [28]
Iran	Bushehr			1423.0	53.0	3.1			43.0		49.0	234	Ardashiriand Hashemi [91]
Malaysia	Selangor	3445–3852		439–880	140–734	2.9–7.7			24–66			75–442	Yap et al. [92]
Malaysia	Shah Alam	4225.3		148.7	31.2				9.0		16.9	30.19	Darus et al. [93]
Nigeria	Ogun State	13.7	254.0	121.0	27.6	855	21.7	21.9	12.7	2.0	41.8	40.9	Olujimi et al. [17]

**Table 4:** The mean concentrations (µg/g) of heavy metals in CACF particles collected from schools in different functional areas.

Element	Urban Schools				Suburban Schools				Residential Schools			
	Minimum	Maximum	Mean	SD	Minimum	Maximum	Mean	SD	Minimum	Maximum	Mean	SD
Fe	6375.0	10,575.0	8650.0	1732.4	6000.0	10,000.0	8166.7	1649.9	7057.3	11,414.5	9438.2	1801.7
Mn	362.5	582.5	481.7	90.7	268.8	438.8	360.8	70.1	247.0	405.4	332.8	65.3
Zn	347.5	559.5	462.3	87.4	236.9	389.9	319.8	63.1	179.7	302.2	246.1	50.5
Pb	171.0	225.0	200.3	22.3	100.0	140.0	120.0	16.3	35.0	55.0	43.3	8.5
Cd	1.6	4.3	3.1	1.1	0.7	2.8	1.8	0.9	0.9	1.9	1.4	0.4
V	60.0	75.0	68.3	6.2	32.0	47.0	40.3	6.2	15.0	30.0	23.3	6.2
Co	8.0	16.0	11.8	3.3	5.5	13.5	9.3	3.3	0.8	7.5	3.3	2.3
Ni	38.0	68.0	53.0	12.2	20.0	50.0	35.0	12.2	4.0	34.0	19.0	12.2
As	9.6	13.0	11.7	1.5	4.8	11.1	8.2	2.6	1.8	5.0	3.9	1.4
Cr	39.0	63.0	52.0	9.9	29.4	48.3	39.7	7.8	21.1	35.6	29.0	6.0
Cu	85.6	136.6	113.3	21.0	65.6	105.9	87.4	16.6	46.6	76.8	63.0	12.5

**Table 5:** The CF and PLI of heavy metals in CACF particles collected from school in different functional areas.

School	CF											PLI
	Fe	Mn	Zn	Pb	Cd	V	Co	Ni	As	Cr	Cu	
Urban schools	0.15	0.51	6.60	16.03	15.33	0.51	0.47	0.71	6.52	0.52	2.06	1.57
Suburban schools	0.15	0.38	4.57	9.60	9.10	0.30	0.37	0.47	4.57	0.40	1.59	1.12
Residential schools	0.17	0.35	3.52	3.47	6.98	0.17	0.13	0.25	2.16	0.29	1.15	0.71
Mean	0.16	0.41	4.90	9.70	10.47	0.33	0.33	0.48	4.42	0.40	1.60	1.15

Notes: CF, contamination factor; PLI, pollution load index.

**Table 6:** Hazard quotient and hazard index of each heavy metal for children in schools of different functional areas.

Risk	Area	Heavy Metals										
		Cr	Ni	Cu	Zn	Cd	Pb	Mn	Fe	Co	V	As
HQ_ing_	Urban schools	1.1E-01	1.6E-02	1.7E-02	9.4E-03	1.9E-02	3.5E-01	6.3E-02	6.3E-03	3.6E-03	5.9E-02	2.4E-01
	Suburban schools	8.1E-02	1.1E-02	1.3E-02	6.5E-03	1.1E-02	2.1E-01	4.7E-02	5.9E-03	2.8E-03	3.5E-02	1.7E-01
	Residential schools	5.9E-02	5.8E-03	9.6E-03	5.0E-03	8.5E-03	7.6E-02	4.3E-02	6.9E-03	1.0E-03	2.0E-02	7.9E-02
HQ_inh_	Urban schools	3.1E-04	4.4E-07	4.8E-07	2.6E-07	5.2E-07	9.7E-06	5.8E-03	6.7E-03	3.5E-04	1.7E-06	6.7E-06
	Suburban schools	2.4E-04	2.9E-07	3.7E-07	1.8E-07	3.1E-07	5.8E-06	4.3E-03	6.3E-03	2.8E-04	9.8E-07	4.7E-06
	Residential schools	1.7E-04	1.6E-07	2.7E-07	1.4E-07	2.4E-07	2.1E-06	4.0E-03	7.3E-03	1.0E-04	5.7E-07	2.2E-06
HQ_derm_	Urban schools	1.0E-02	9.6E-05	9.2E-05	7.5E-05	3.0E-03	3.7E-03	2.6E-03	1.2E-03	7.2E-06	9.5E-03	9.3E-04
	Suburban schools	7.7E-03	6.3E-05	7.1E-05	5.2E-05	1.8E-03	2.2E-03	1.9E-03	1.1E-03	5.7E-06	5.6E-03	6.5E-04
	Residential schools	5.7E-03	3.4E-05	5.1E-05	4.0E-05	1.4E-03	8.1E-04	1.8E-03	1.3E-03	2.0E-06	3.2E-03	3.1E-04
HI	Urban schools	1.2E-01	1.6E-02	1.7E-02	9.5E-03	2.2E-02	3.5E-01	7.1E-02	1.4E-02	4.0E-03	6.9E-02	2.4E-01
	Suburban schools	8.9E-02	1.1E-02	1.3E-02	6.6E-03	1.3E-02	2.1E-01	5.3E-02	1.3E-02	3.1E-03	4.1E-02	1.7E-01
	Residential schools	6.5E-02	5.8E-03	9.7E-03	5.0E-03	9.9E-03	7.6E-02	4.9E-02	1.6E-02	1.1E-03	2.4E-02	7.9E-02
RfD_ing_		3.0E-03	2.0E-02	4.0E-02	3.0E-01	1.0E-03	3.5E-03	4.7E-02	8.4E+00	2.0E-02	7.0E-03	3.0E-04
RfD_inh_		2.9E-05	2.1E-02	4.0E-02	3.0E-01	1.0E-03	3.5E-03	1.4E-05	2.2E-04	5.7E-06	7.0E-03	3.0E-04
RfD_derm_		5.0E-05	5.4E-03	1.2E-02	6.0E-02	1.0E-05	5.3E-04	1.8E-03	7.0E-02	1.6E-02	7.0E-05	1.2E-04

**Table 7:** LCR of each heavy metal for children in schools of different functional areas.

Risk	Area	Heavy Metals					
		Cr	Ni	Cd	Pb	Co	As
LCR_ing_	Urban schools	1.3E-04	2.7E-04	1.2E-04	1.0E-05	7.1E-04	1.1E-05
	Suburban schools	1.0E-04	1.8E-04	7.0E-05	6.2E-06	5.6E-04	7.6E-06
	Residential schools	7.4E-05	9.7E-05	5.4E-05	2.2E-06	2.0E-04	3.6E-06
LCR_inh_	Urban schools	3.7E-09	7.6E-09	3.3E-09	2.9E-10	2.0E-08	3.0E-10
	Suburban schools	2.8E-09	5.0E-09	2.0E-09	1.7E-10	1.6E-08	2.1E-10
	Residential schools	2.1E-09	2.7E-09	1.5E-09	6.3E-11	5.6E-09	1.0E-10
LCR_derm_	Urban schools	2.1E-07	4.3E-07	1.9E-07	1.7E-08	1.1E-06	1.7E-08
	Suburban schools	1.6E-07	2.9E-07	1.1E-07	1.0E-08	8.9E-07	1.2E-08
	Residential schools	1.2E-07	1.6E-07	8.6E-08	3.6E-09	3.2E-07	5.7E-09
TLCR	Urban schools	1.3E-04	2.7E-04	1.2E-04	1.0E-05	7.1E-04	1.1E-05
	Suburban schools	1.0E-04	1.8E-04	7.0E-05	6.2E-06	5.6E-04	7.6E-06
	Residential schools	7.4E-05	9.8E-05	5.4E-05	2.3E-06	2.0E-04	3.6E-06
Sf		4.2E-01	8.4E-01	6.3E+00	8.5E-03	9.8E+00	1.5E-01
